# Vitamin C and epigenetics: A short physiological overview

**DOI:** 10.1515/med-2023-0688

**Published:** 2023-06-22

**Authors:** Voja Pavlovic, Milan Ciric, Milan Petkovic, Mladjan Golubovic

**Affiliations:** Department of Physiology, Medical Faculty University in Nis, Bulevar Dr. Zorana Djindjica 81, 18000 Nis, Serbia; Clinic for Cardiosurgery, Department of Anesthesiology and Intensive care, University Clinical center of Nis, 18000 Nis, Serbia; Department of Physiology, Medical Faculty University in Nis, 18000 Nis, Serbia

**Keywords:** ascorbic acid, epigenetics, DNA methylation, histone modification

## Abstract

In recent years, ascorbic acid (vitamin C) has acquired great interest due to its multiple functions, which results in homeostasis of normal tissues and organs. On the other hand, it has been shown that epigenetic modifications may have an important role in various diseases and therefore are a focus of the extraordinary investigation. Ascorbic acid serves as a cofactor for ten-eleven translocation dioxygenases, which are responsible for deoxyribonucleic acid methylation. Also, vitamin C is required for histone demethylation, since it acts as a cofactor of Jumonji C-domain-containing histone demethylases. It seems that vitamin C may be a mediator between the environment and the genome. The precise and multistep mechanism of ascorbic acid in epigenetic control is still not definitely determined. This article intends to provide the basic and newly discovered functions of vitamin C that are related to epigenetic control. Also, this article will help us to better understand the functions of ascorbic acid and will provide the possible implications of this vitamin in the regulation of epigenetic modifications.

## 1 Introduction

Ascorbic acid, also known as vitamin C or ascorbate, is an essential vitamin that has different key roles in human physiology. All mammals are able to synthesize vitamin C in the liver and provide a steady supply of vitamin C to the body through circulation. However, primates, guinea pigs, and fruit bats have lost this capability due to mutations in the gene encoding l-gulonolactone oxidase enzyme [1]. This enzyme is responsible for catalyzing the conversion of l-gulono-g-lactone into water-soluble ascorbic acid [2]. Consequently, humans must acquire vitamin C from dietary sources to maintain tissue concentration. Low dietary vitamin C intake (with plasma ascorbic acid concentrations <11 µmol/l) results in a vitamin C deficiency disease named “scurvy,” a potentially fatal disease in humans and other animals unable to endogenously produce ascorbic acid [3]. Nowadays, scurvy is very rare but hypovitaminosis of ascorbic acid (with plasma ascorbic acid concentrations <23 µmol/l) is frequent in the adult population [4] and is more dominant among subgroups of the pregnant woman and smokers [5].

Ascorbic acid is an electron donor (possesses an intensive reductive potential); it can be oxidized to the ascorbic radical and then to dehydroascorbic acid. Ascorbic radical is a relatively stable and unreactive molecule [6]. On the other hand, dehydroascorbic acid is an unstable molecule with a short half-life [2], and it is rapidly reduced back to ascorbic acid by glutathione-dependent cellular reducing systems [3]. Both forms of vitamin C (ascorbic and dehydroascorbic acid) are present in the human diet, and their absorption takes place at the level of enterocytes of the small intestine. By entering into the cells, vitamin C displays key roles in many biological processes acting as an electron donor. Micromolar concentrations of vitamin C exert redox function and can reduce dangerous reactive oxygen species (ROS), which are produced during the normal metabolic mitochondrial oxidative metabolism [7]. However, pharmacological or millimolar vitamin C concentrations may act as pro-oxidant and exert a cytotoxic effect against tumor cells compared to normal cells [8]. Besides its antioxidative functions, vitamin C acts as a cofactor for enzymes involved in the process of collagen synthesis [9], functions as a reducing agent for several iron-dependent enzymes, and affects iron metabolism [10]. Among different biological functions of vitamin C, relatively the newly discovered role of vitamin C is that it acts as a cofactor of the ten-eleven translocation (TET) and Jumonji C-domain-containing histone demethylases (JHDM), which belong to the Fe(ii)- and α-ketoglutarate dioxygenase superfamily, which are involved in deoxyribonucleic acid (DNA) methylation and histone modification [7,11].

Given the diverse roles of vitamin C in human physiology, the purpose of this article is to clarify the role of vitamin C in epigenetic regulation, the role of vitamin C in modulating the activity of some enzymes involved in epigenetic reprogramming, and the potential role of vitamin C in the outcome of epigenetic therapy.

## 2 Basic biological functions of vitamin C

To apply its biological properties, vitamin C must enter into the cells by using specific transporters. Since vitamin C can exist in two different forms (ascorbic acid and dehydroascorbic acid), there are two dissimilar transport mechanisms. Ascorbic acid is co-transported, across the cell membrane, through sodium-dependent vitamin C transporters 1 and 2 (SVCT1 and SVCT2) together with sodium [12]. SVCT1 transporters are mainly expressed at the level of intestinal epithelial and renal tubular cells, and they are responsible for the dietary absorption of ascorbic acid [13]. On the other hand, SVCT2 transporters are widely expressed in various tissues and are responsible for the distribution of ascorbic acid from the bloodstream to target organs [14]. However, the absorption of dehydroascorbic acid from the intestinal lumen occurs primarily by facilitated glucose transporters 2 and 8 (GLUT2 and GLUT8) [15]. Dehydroascorbic acid is able to compete with dietary glucose for uptake into the cells through GLUT1, GLUT3, and GLUT4 [6]. In the cells, dehydroascorbic acid is rapidly reduced to ascorbic acid and stays trapped intracellularly [6]. During physiological conditions, dehydroascorbic acid is present in small amounts, compared to glucose, which results in a minor dehydroascorbic acid uptake via GLUT transporters [15].

In humans, the recommended daily intake of vitamin C is 75–90 mg/day [8] and should be achieved with a diet containing fruits and vegetables. However, for elderly people who are at risk of developing cardiovascular diseases and cancer as well as those with reduced immune function, higher amounts of vitamin C (120 mg/day) have been recommended [16]. Also, vitamin C in human body shows a diverse distribution (Table 1). Plasma concentration of vitamin C is tightly controlled by intestinal uptake and kidney excretion [6]. Additionally, all intracellular vitamin C concentrations are higher than its extracellular concentrations, omitting the erythrocytes that have vitamin C concentrations similar to those in plasma [6]. The highest vitamin C concentrations are found in the brain and adrenal glands [12], while circulating blood cells (neutrophils, lymphocytes, monocytes, and platelets) have vitamin C concentrations ranging between 1 and 4 mM [17].

**Table 1:** Tissue distribution of vitamin C in some tissues of human body

Tissue	Vitamin C concentration (mM)
Heart	0.2–0.4
Adrenal glands	4–10
Kidney	0.3–0.5
Lungs	1
Skeletal muscle	0.2–0.4
Liver	1
Brain	2–10

Vitamin C displays diverse functions in the human body. As an electron donor, it can reduce ROS and therefore protects and maintains protein integrity in cells intracellularly and extracellularly [6] against various mutations [1]. Besides its antioxidative functions, vitamin C influences iron metabolism by stimulating ferritin synthesis and inhibiting ferritin degradation as well as the absorption of ferric iron (Fe(iii)) [10]. Similarly, for the absorption of iron from the intestinal lumen, ferric iron has to be reduced to the ferrous state (Fe(ii)), which occurs in the presence of vitamin C [18]. In addition, vitamin C is an essential cofactor for enzymes (prolyl-3-hydroxylase, prolyl-4-hydroxylase, and lysyl hydroxylase) involved in the hydroxylation of proline and lysine, which elucidates the crucial role of this vitamin in collagen synthesis [9]. Also, ascorbic acid promotes the reduction of iron to the ferrous state and accordingly keeps prolyl hydroxylase active [19]. Reduced amount of vitamin C results in decreased collagen stability and finally results in the development of scurvy [6]. It has been shown that vitamin C stimulates the activity of natural killer cells and cytotoxic T lymphocytes [20]. Furthermore, earlier studies showed that vitamin C inactivated DNA and RNA viruses with attenuated viral activity [21,22]. In line with this, the results of the same studies showed that vitamin C is a vital factor in the production of interferon type I during antiviral response [22] and is present in the respiratory tract where it helps to alleviate symptoms of upper respiratory tract infections [23].

Among the diverse biological functions of vitamin C, a relatively newly discovered role of this vitamin is to act as a modulator of the activity of several (including TET and JHDMs) enzymes, which shows its essential role in epigenetic regulation. Also, a recent report documented the role of vitamin C in regulating the function of embryonic stem cells and hematopoietic stem cells from epigenetic alterations [24], indicating the ability of vitamin C to modulate the epigenome with some potential therapeutic benefits.

## 3 Vitamin C and DNA methylation

Various reports documented that normal epigenetic regulation is altered during tumorigenesis and disrupted DNA methylation is a hallmark of cancer [25], which leads to genomic instability and malignant transformation [26]. Methylation of DNA cytosines represents epigenetic modification that regulates gene expression, without changing DNA sequence, and simultaneously limits the accessibility of transcription factors to promoters or any other regulatory component [6]. Most of the methylated cytosines are located in heterochromatic regions, making certain that chromatin stays closed and gene silenced when cells do not have the capacity to be actively transcribed [3]. Therefore, under physiological conditions, around 80% of cytosines are methylated in the mammalian genome, in the context of CpG dinucleotides [27]. On the other hand, hypomethylation and hypermethylation are related to genomic instability and carcinogenesis. By hypermethylation of CpG-rich regions (also known as CpG islands) in promoter regions, DNA methylation is able to directly silence gene expression [28]. Since around 70% of human gene promoters have associated CpG islands, this is a crucial mechanism by which DNA methylation controls gene expression [29]. In line with this, DNA hypomethylation can lead to the expression of atypical oncogenes and further tumorigenesis, while DNA hypermethylation may result in a silenced expression of DNA repair genes and reduced DNA repair proteins [30,31]. Therefore, making the balance between writers and erasers of DNA methylation preserves cells from obtaining indefinite changes to the genetic code.

During the process of DNA methylation, DNA methyltransferases (DNMT1, 3A, and 3B) catalyze the addition of methyl group to C5 of cytosine, forming 5-methylcystosine (5mC), which is the key step in the DNA methylation process [32]. Most of the CpG islands are close to gene promoter regions, and they are the most methylated regions in the mammalian genome [29]. Usually, DNMT1 is responsible for the preservation of methylation during DNA replication, while DNMT3A and DNMT3B are essential for de novo synthesis [25]. Furthermore, the methylation of lysine, by histone lysine methyltransferases, is an essential histone modification, and it is demethylated by histone lysine demethylases [33]. Vitamin C acts as a cofactor for TET enzymes (TET1, 2, and 3) that catalyze the oxidation of 5mC to 5-hydroxymethylcytosine (5hmC). Demethylation process continues with the oxidation of 5hmC to 5-formylcytosine (5fC) and 5-carboxylcytosine (5caC), which are finally removed and replaced with unmethylated cytosine through the base excision repair mechanism [34]. Embryonic stem cells undergo a stable 5hmC modification, which can vary widely in different cells. At the same time, the presence of 5hmC results in a passive loss of DNA methylation, since DNMT1 is not able to recognize hemimethylated 5hmC [35]. Levels of 5fC and 5caC represent the rare modifications in genome, and their frequency is minor [25].

Vitamin C is responsible for TET activity, as a necessary step during DNA methylation and the concentration of TET enzymes corresponds with DNA methylation status. The involvement of TET1 in the oxidation of 5mC to 5hmC was documented back in 2009 [36], while the roles of TET2 and TET3 in the same process were reported later [37]. Dysregulation of DNA methylation may be the result of several mechanisms, including the reduced activity of TET enzymes, or DNMT, which leads to hypomethylation (loss of oncogene silencing) or hypermethylation (aberrant silencing of different tumor suppressors) (Table 2). These TET enzymes are Fe(ii) and 2-oxoglutarate-dependent dioxygenases. It is well documented that reduced levels of 5hmC are found in different human cancers, indicating a loss of TET activity [38]. Besides a decrease in TET expression and mutational aberrations, decreased levels of 5hmC may be due to inadequate amounts of TET cofactors, including vitamin C and oxygen, which are common in the hypoxic tumor microenvironment [16]. In accordance with previous findings, the addition of vitamin C enhanced DNA demethylation, by increasing TET activity in embryonic stem cells [39] and vitamin C induces widespread DNA demethylation of around 2,000 genes in embryonic stem cells [40]. In addition, TET2 mutations are linked to increased 5mC and reduced 5hmC in patients with myeloid malignancies [41]. A recent study demonstrated that the oral supplementation of vitamin C resulted in an increased 5hmC/5mC ratio compared to placebo-treated patients [42]. In human skin cancer cells, the application of vitamin C, against UV-mediated apoptosis, showed demethylation and reactivation of silenced tumor suppressor genes p21 and p16 in a TET-dependent manner [43]. *In vitro* study confirms that vitamin C induces an 8-fold increase of hydroxymethylation, while other reducing agents (vitamin E, vitamin B1, NADPH) could not stimulate the TET2 catalytic activity [44]. However, another study reported that some reducing agents are able to stimulate TET activity but without such efficiency as vitamin C [45], highlighting the role of vitamin C in promoting TET activity. Together with vitamin C, oxygen is also necessary for the proper activity of TET enzymes. Previous findings showed that TET activity is reduced by tumor hypoxia in different cells [46]. Hypoxic tumor tissue shows hypermethylation at tumor suppressor gene promoters, suggesting that, besides vitamin C, oxygen is one of the main regulation factors of DNA methylation.

**Table 2:** Role of TET enzymes in general development

Enzyme	General role in development
TET1	Highly expressed in mouse embryonic stem cells where it preserves them in pluripotent stem state; necessary for brain development; lack of this enzyme leads to memory disorder and altered ectoderm development
TET2	Necessary for brain development and hematopoietic development; lack of TET1 leads to exencephaly; loss of this enzyme results in leukemia initiation and other hematopoietic disorders
TET3	Crucial for proper embryonic development, neural and mesoderm cell growth and brain development; loss of this enzyme leads to embryonic lethality intellectual disorders; lack of TET1 enzyme results in holoprosencephaly

Even in the last few years, many research studies have been conducted about the relationship between vitamin C and TET activity. In the context of leukemia research, the precise role of vitamin C in TET catalytic activity still remains unclear. Up to now, there are two different theories that are able to explain the potential relationship between vitamin C and TET activity. The first mechanism includes the possibility that TET enzymes have a specific binding site for vitamin C where it reduces enzyme-bound iron. Another possibility is that vitamin C induces TET activity not through the binding site but rather by converting (Fe(iii)) to (Fe(ii)). Further analysis is required to clarify whether the exact mechanism is responsible for TET activity. Furthermore, the role of vitamin C for optimal TET activity requires adequate amounts of iron in Fe(ii) form for optimal 5hmC production [46]. In line with this, the same report documented that 100-fold less Fe(ii) (instead of Fe(iii)) is sufficient to sustain the full TET activity, while iron activity is mainly dependent on pH [46]. Also, oxygen and α-ketoglutarate are necessary for optimal TET activity [47]. On the other hand, taking into account that vitamin C levels are markedly reduced in pregnant women [5] and cancer patients, they need additional supplementation, especially for the elderly (aged 75 years) [6]. This is in line with previous reports that show a strong correlation between the reduction in vitamin C (decreased absorption and reduced cellular uptake) and the process of aging, showing that vitamin C levels reduced around 50% of leukocytes at the age of 85 [11], which shows that vitamin C may have an influence on the development of neurodegenerative disorders [11]. The earlier report demonstrated that ascorbic acid has a key role in preventing aging in embryonic stem cells *in vitro* [48]. Similarly, in mesenchymal stem cells, vitamin C increases telomerase activity and gene expression that protect the telomere stability [48]. Moreover, in normal conditions, DHA is rapidly intracellularly converted to ascorbate. However, if this mechanism is overloaded, it may lead to the degradation of vitamin C as the result of the short half-time of DHA [49].

## 4 Vitamin C and histone modification

Besides DNA methylation, the transfer of a methyl group of residues of histone proteins is also a key epigenetic mark that controls stem cell homeostasis. The addition of methyl group to lysine residues is catalyzed by histone methyltransferases (HMTs) [50]. In DNA methylation, only one methyl group is added to cytosine, but in histone methylation, two or three methyl groups may be added to lysine [51]. Recently, the process of histone methylation was considered to be irreversible. However, the earlier report documented the removal of the methyl group from the methylated histone, indicating that the histone methylation process is reversible [52]. The majority of HMTs are JHDMs, which can catalyze demethylation that requires Fe(ii) and α-ketoglutarate [33]. Until now, around 20 different proteins that belong to the JHDM, with a catalytic activity to demethylate histones, have been discovered [53]. The regulatory roles of these enzymes are dependent on vitamin C for their optimal catalytic activity [54]. This observation was confirmed by various reports, which documented that vitamin C is necessary for regular T-cell maturation [55], neural differentiation of embryonic stem cells [56], hearing and functions [57], and cardiac development [58]. Similar findings were confirmed by using *in vitro* study where the depletion of vitamin C resulted in almost complete inhibition of histone demethylation [59]. Based on the abovementioned observations, the role of vitamin C in epigenome regulation needs further consideration.

## 5 Conclusion

Taken together with previous findings, indicating the role of vitamin C for the regular function of TET and JHDM enzymes and the resulting DNA and histone demethylation, the lack of this water-soluble vitamin may probably result in weakness of cellular reprogramming, which may result in neoplastic growth, especially among hematological cancers. Further examinations are necessary to confirm whether vitamin C supplementation may result in increased catalytic activity of epigenetic regulators. Having in mind that atypical epigenetic regulation is very common in almost all cancers, the regulatory effect of vitamin C on the methylation of DNA and histones may have some benefits in some types of neoplastic disorders.
